# Prospective observation of *Clostridium histolyticum* collagenase for the treatment of Dupuytren’s disease in 788 patients: the Austrian register

**DOI:** 10.1007/s00402-019-03226-3

**Published:** 2019-07-17

**Authors:** Arora Rohit, Angermann Peter, Aspalter Paul, Binter Anja, Deml Christian, Danninger Renate, Gärner Stefan, Hager Dietmar, Jeschke Johannes, Kaiser Peter, Keller Marco, Leixnering Martin, Neuwirth Maximilian, Pezzei Christoph, Schmidle Gernot, Schmölzer Gerald, Steirer Tobias, Wlk Matthias, Zadra Armin, Gabl Markus

**Affiliations:** 1 Department of Trauma Surgery, Medical University of Innsbruck, Anichstr. 35, 6020 Innsbruck, Austria; 2 Krankenhaus Amstetten, Amstetten, Austria; 3 Klinikum Klagenfurt Am Wörthersee, Klagenfurt, Austria; 4 Sanatorium Kettenbrücke, Innsbruck, Austria; 5 Landeskrankenhaus Vöcklabruck, Vöcklabruck, Austria; 6 Privatklinik Döbling, Wien, Austria; 7 Diakonissen Klinik Linz, Linz, Austria; 8 Privatklinik Maria Hilf, Klagenfurt, Austria; 9 Kantonspital Baselland, Basel, Switzerland; 10 Rudolfingerhaus Wien, Wien, Austria; 11 Lorenz Böhler Unfallkrankenhaus, AUVA, Wien, Austria; 12 Krankenhaus Steyr, Steyr, Austria; 13 Ordination Im Zentrum, Furth Bei Göttweig, Austria; 14 Orthozentrum Wien Hietzing, Wien, Austria; 15 Ordination Dr Zadra, Strass, Austria

**Keywords:** Dupuytren, Collagenase, Xiapex, Complication, Adverse event, Fibromatosis

## Abstract

**Introduction:**

Since March 2011, the microbial collagenase of *Clostridium histolyticum (*Xiapex®, Swedish Orphan Biovitrum AB, Stockholm, Sweden) has become available in the European Union for treatment of Dupuytren’s disease. The purpose of this study was to evaluate potential safety risks of Xiapex® and to contribute to a better understanding for its use.

**Methods:**

A prospective, non-interventional, observational study using Xiapex® for Dupuytren’s disease named XIANIS was conducted between 1.10.2011 and 01.10.2017. Treatment was conducted in accordance to the manufacturer information. Patients were invited for follow-up after 1 week, 1 month, 3 months and 1 year. Demographic data, treatment data, pain levels, anaesthetic application during passive manipulation, subjective function improvement, subjective satisfaction and adverse events were recorded.

**Results:**

788 patients with 814 treatments were included who suffered from Dupuytren’s contracture for a mean of 64 months. The metacarpophalangeal joint was affected in 57% of cases and the PIP joint in 40.8% with a mean contracture of 39° and 56°, respectively. A change in the contracture down to 0°–5° was reported in 66.5% of cases, while 25.5% achieved a partial improvement. The pain during the injection was rated 4.5 and 3.3 during passive manipulation. Adverse events were reported in the majority of treated patients with skin tears being one main common event (26%). Further adverse outcomes were bleeding/hematoma, joint swelling, injection-site swelling, pressure sensitivity, erythema, injection-site pain, peripheral edema, blood blisters, blisters, painless lymphadenopathy, painful lymphadenopathy, axillary pain, arthralgia and sensory abnormality. There were no reported tendon ruptures, anaphylactic reactions or ligament injuries. On 1-year follow-up, 29% showed an increased contracture of a mean of 24° with the need for surgical treatment in 2% of patients. 74% of patients were very satisfied and 72% showed a high functional improvement.

**Conclusion:**

The injectable collagenase *Clostridium histolyticum* (Xiapex®) proved to be effective and safe in patients with Dupuytren’s disease. Minor adverse events disappeared within 30 days and the need for surgical treatment within 1 year was very low (2%). No major complications or rare side effects were seen in this prospective observational study.

## Introduction

Dupuytren’s disease is a common benign fibromatosis of the fibrous skeleton of the hand manifesting in a progressive flexion contracture of the hand [1]. This disease is more common in people from northern Europe than in Asian and African people with a higher incidence in older and male persons [2]. Alcohol, nicotine and work with vibrating machines seem to have a negative influence on the course of the disease [2]. There is no known cure for this disorder [3, 4]. Therefore, treatment options target primarily on restoring the normal function of the hand by disrupting or removing the Dupuytren’s cord but not resolving the disease.

Surgical treatment options include complete or partial fasciectomy, dermofasciectomy, fasciotomy or percutaneous needling [3, 5, 6]. The complication rate for surgical treatment ranges between 4 and 39% including poor wound healing, scar pain, paraesthesia, hypoesthesia, flare reaction, complex regional pain syndrome, infections, hematoma and nerve or vessel injuries [7–10]. Until recently, nonsurgical interventions have proved to be largely ineffective and rejected clinically [11].

Since March 2011, the microbial collagenase of *Clostridium histolyticum (*Xiapex®, Swedish Orphan Biovitrum AB, Stockholm, Sweden) has become available in the European Union as the first injectable drug treatment option for Dupuytren’s disease.

Its marketing authorization origins from the results of two randomized, placebo-controlled, double-blinded studies—CORD (Collagenase Option for the Reduction of Dupuytren’s) I and CORD II [12]. Treatment was judged successful if the flexion contracture was reduced to 0°–5°. In the CORD I study, 64% of the patients in the collagenase arm achieved this stringent criterion compared to only 7% of the patients in the placebo group. 85% of the patients in the same study had a significant improvement of the contracture of at least 50%. The CORD II study as well as further clinical studies confirmed the efficacy and safety of this investigational treatment option [13–19, 4, 20–22].

Although proof of efficacy and safety has been impressively provided, many clinical studies include only a relatively low number of patients. This could prevent detecting very rare serious adverse events. Moreover, clinical studies like the CORD I and II studies do not always reflect outcomes of the real-world setting because of its strict study design. As an example, local anaesthesia on day 2 was not permitted for patients, because of a potential interaction with the collagenase that had to be ruled out.

At the time Xiapex® was introduced in clinical routine in Austria, there were few surgeons in Europe experienced in applying this non-surgical technique. Since the data with European patients were scarce, the Medical University of Innsbruck and the Austrian Society of Hand Surgery decided to conduct a prospective non-interventional, observational multicenter study under real-life conditions to assess the efficacy and safety of Xiapex® in the daily routine.

The purpose of this study was to reveal potential safety risks of Xiapex®, if any, and to contribute to a better understanding for its use.

## Materials and methods

A prospective, non-interventional, observational study using Xiapex® (Swedish Orphan Biovitrum AB, Stockholm, Sweden) for Dupuytren’s disease named XIANIS was conducted between 1.10.2011 and 01.10.2017 in Austria following the Austrian drug law (AMG §2 Abs. 3). The collection of patient’s data was approved by local ethic committee of the Medical University of Innsbruck. In general, non-interventional studies in Austria allow only those procedures that are completely in line with the common clinical practice and exclude any deviations from the routine which might lead to additional liabilities for patients. Therefore, mandatory follow-up (FU) visits as well as additional diagnostic or therapeutic measures were not permitted.

The Austrian Society of Hand Surgery decided that Xiapex® should only be administered by trained hand surgeons for patient’s safety and for registration of any complications and side effects in a centralized data base.

Physicians were required to enter treatment data for each single patient into a password-protected, online database questionnaire (www.xianis.at, Craft And Value) after each medical contact. Passwords were generated automatically by the system and the access to the database was granted by the principal investigator. 24 physicians working at 17 different facilities participated in this evaluation.

Patients older than 18 years who suffered from Dupuytren’s disease were included if the contracture was between 20° and 100° for the metacarpophalangeal (MCP) joint and between 20° and 80° for the proximal interphalangeal (PIP) joint. The decision for a treatment with Xiapex® or a different surgical or non-surgical treatment was made in advance before study inclusion and based on patients’ wishes and surgeons’ recommendations. All patients gave their written informed consent for study inclusion.

The course of treatment was in accordance with the manufacturer information. After 58 mg Xiapex was injected into the cord, patients were observed for 30 min to exclude any risk of potential systemic adverse reactions. Patients were either treated on an outpatient basis or stayed overnight depending on the hospital’s policy.

24 h after the injection (day 2 FU), the treated fingers were passively extended for 10–20 s to support the breakage of the cord. It was recorded if a single or multiple manipulations were needed for breakage. Passive extension was conducted either under local anaesthesia, a nerve block or a plexus block.

Patients were invited for further FU after 1 week (day 7 FU), 1 month (day 30 FU), 3 months (day 90 FU) and 1 year (day 365 FU).

Patients were included on the day of the Xiapex injection and the following data were recorded: age, gender, previous treatment (subdivided in fasciectomy, dermofasciectomy, needling, fasciotomy, other unspecified surgical treatments and conservative non-surgical treatment), duration of disease, finger involvement, the global (MCP + PIP) degree of contracture as well as for the MCP and PIP joint, pain level during injection and any adverse event.

The pain level [using the visual analogue scale (VAS)], the degree of contracture and any adverse events were also recorded for the day 2, day 7, 30, 90 and 365 FU.

The method of anaesthesia (subdivided in local anaesthesia, nerve block and plexus anaesthesia) was recorded for the day of manipulation.

Subjective functional improvement and satisfaction of the patients were recorded for the day 30 and 365 FU. Both parameters were assessed by a simple question: “Do you have any functional improvement by the therapy” and “Are you satisfied with the therapy”. Answers were subdivided into “high”, “intermediate” or “no improvement” for the functional assessment and “very”, “partially” or “not satisfied” for the satisfaction assessment.

The need for additional surgical treatment was recorded for the days 30 and 365 FU.

A change in the contracture to an absolute extension deficit of 0°–5° was recorded as a complete improvement. A change to an absolute deficit of more than 5° was recorded as a partial improvement independent of the amount of achieved correction. Just cases with no objectively measurable changes were recorded as no change after manipulation. A recurrence was defined as a newly increased contracture of relatively 20° in comparison to the state after manipulation.

All data are presented using descriptive statistics.

## Results

A total of 788 patients (87% male; 13% female; mean 63.5 years (range 25–90 years) with 814 injections were included in the collagenase database. 97% of all treated patients completed day two visits, 90% the subsequent day 7 medical follow-up, 78% day 30, 64% day 90 and 32% day 365 follow-up.

Seven percent of patients had a previous treatment including fasciectomy (*n* = 38), dermofasciectomy (*n* = 4), needling (*n* = 3), fasciotomy (*n* = 2), other unspecified surgical treatments (*n* = 4) and conservative non-surgical treatment (dynamic splinting *n* = 3, radiotherapy *n* = 2). Sixty-seven percent of patients were treated in an outpatient hospital setting while 26% were treated in a stationary setting and 3% in a private practice. Data for 30 patients were missing.

The patients suffered from Dupuytren’s contracture for a mean of 64 months (range 3–350 months). The small finger was the most affected finger (43.6%) followed by the ring finger (39.8%), the middle finger (11.8%), the thumb (2.5%) and the index finger (2.3%).

The metacarpophalangeal joint was affected in 57% of cases, the PIP joint in 40.8% and the DIP joint in 2.2%. A mean of 1.8 cords were treated per person.

The degree of contracture is shown in Table 1.

**Table 1:** Mean and range of Dupuytren contracture

	Before treatment	Day 2 FU	Day 7 FU	Day 30 Fu	Day 90 FU	Day 365 FU
MCP + PIP contracture (global contracture)	64.2° (10°–170°)	21° (5°–100°)	19.9° (5°–95°)	19.1° (5°–95°)	18.0° (3°–80°)	26.2° (5°–70°)
MCP joint contracture	39° (10°–100°)	13.9° (5°–70°)	14.7° (3°–80°)	18.3° (5°–80°)	17.5° (3°–110°)	15.6° (5°–30°)
PIP joint contracture	56.0° (5°–110°)	19.6° (5°–75°)	19.1° (4°–80°)	18.0° (5°–80°)	19.4° (3°–80°)	20.7° (5°–50°)

A change in the contracture down to 0°–5° was reported in 66.5% of cases, while 25.5% achieved a partial improvement (change in the contracture down to more than 5° extension deficit) and 8% showed no change after manipulation.

An extension manipulation on day 2 was performed using local anaesthesia in 61.3% of patients, nerve block in 30.3%, plexus anaesthesia 2.4% and no data were acquired in 6.1%.

A spontaneous cord rupture was seen in 5.8% of patients. Only one passive extension manipulation was needed in 46.5% of patients while 46.8% needed multiple manipulations.

The pain during the injection was rated 4.5 (range 0–10) and 3.3 (range 0–10) during passive manipulation. Patients reported only a minor discomfort during further follow-up (1.6 on day 7, 1.3 on day 30, 1.1 on day 90 and 1.1 on day 365).

**Fig. 1:**
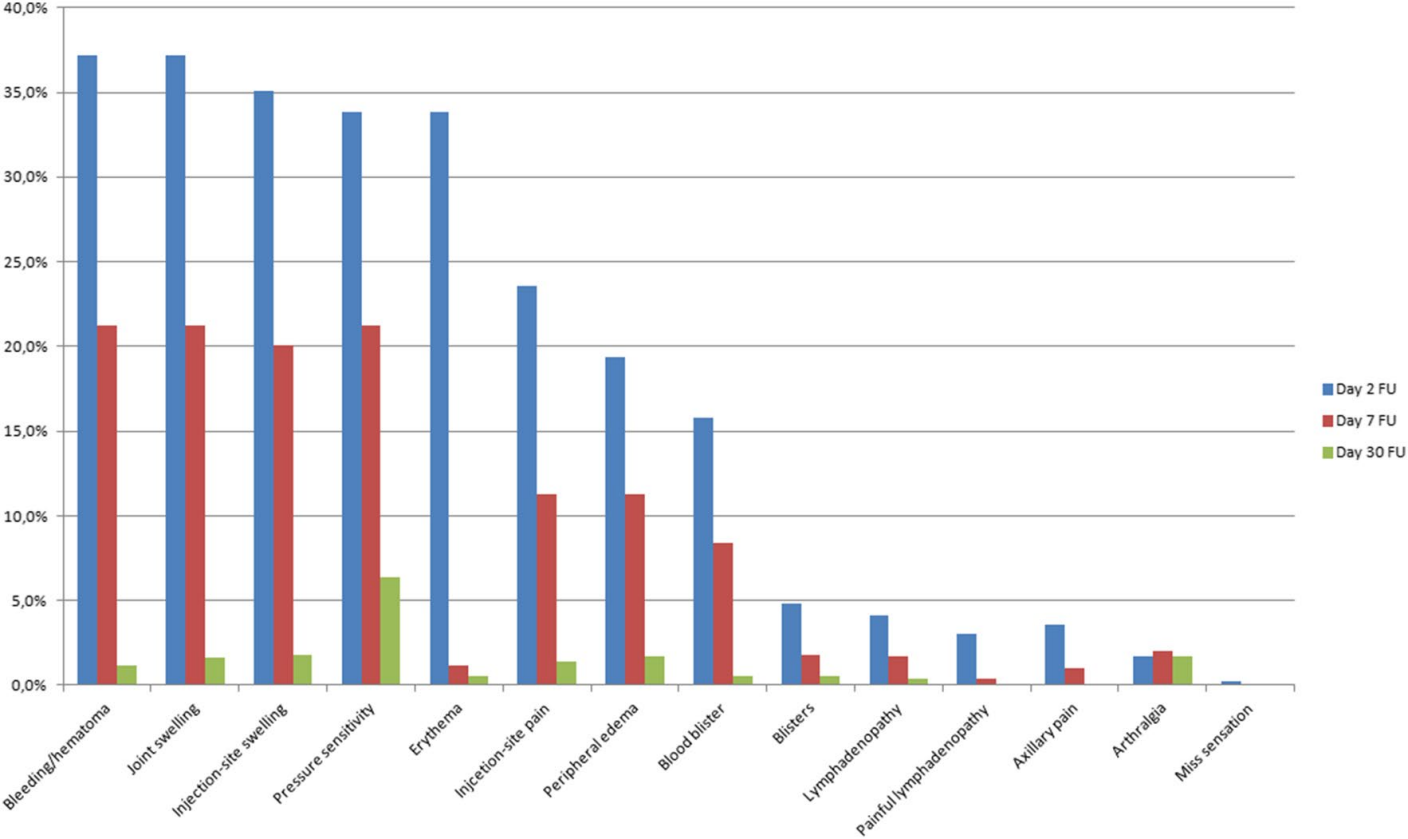
Adverse events on follow-up days 2, 7 and 30

Adverse events were reported in the majority of treated patients with skin tears being one main common event (26%). The size of the skin tears was less than 5 mm in 58.9%, between 5 and 15 mm in 36.4% and above 15 mm in 4.6% of patients. The frequency of further adverse outcomes is shown in Fig. 1. There were no reported tendon ruptures, anaphylactic reactions or ligament injuries.

At 365-day FU, 71% of patients who returned for follow-up did not show an increased contracture. The remaining 29% showed a mean contracture increase of 24°. At 30-day FU and 365-day FU only 1% and 2%, respectively, needed a surgical treatment. The main rational for surgical treatment was a symptomatic patient with complains because of a contracture recurrence with at least an absolute 20° extension deficit.

The subjective patient satisfaction was mostly very high (Table 2).

**Table 2:** Subjective improvement and satisfaction of the patients

	Day 7 FU (%)	Day 30 FU (%)	Day 365 FU (%)
Functional improvement			
High improvement	78	80	72
Intermediate improvement	17	14	19
No improvement	5	6	10
Satisfaction			
Very satisfied	85	86	74
Partially satisfied	13	13	22
Not satisfied	2	1	4

## Discussion

This prospective observational study in a large cohort showed that the application of Xiapex® for Dupuytren’s disease is effective and safe as adverse events were of mainly minor genesis, resolved within a short time period and Xiapex® did not disturb or complicate potential surgical treatment in the future.

The effectiveness of Xiapex® has been shown in multiple previous studies [23, 18, 17, 24, 14, 25, 13] with 51–92% of patients achieving a postinterventional contracture of 0°–5° directly after manipulation [13, 25, 17, 26] similar to our observational report with 66.5%.

Minor adverse side effects were observed similar to literature [11, 27–29, 14]. The main ones were swelling, hematomas, blisters, pressure sensitivity and skin rupture on day 2. However, most of these side effects were transient and were not present at the 30-day FU. An increase of adverse events can be seen if multiple joints are treated in the same hand simultaneously using an increased medication dose [30]. However, these data were acquired during initial explorative investigations. Potentially, this study could just see transient adverse events because treatment was just conducted on a single finger and a limited dose of collagenase according to the protocol. In addition, it was shown that multiple effected joints can be treated concurrently without safety risks and with a high patient satisfaction [29–31]. Although tendon ruptures were reported as a serious complication following collagenase injections [14, 30], they were not seen in our prospective observation. The reason might be, that injections were performed just by hand surgeons and that this complication was already known and care was taken to apply the collagenase only into the Dupuytren cord and not into the tendon.

Similar to literature, skin tears occurred in a quarter of patients with an uneventful healing [27, 29, 16, 32]. The average healing time was 11 days independent from the dressing [32]. The risk for skin lacerations seems higher for worse initial contractures, MCP joint involvement bilateral disease, previous surgical treatment [32] and treatment for two affected joints concurrently [29].

Collagenase treatment was shown to potentially be a painful process with a mean VAS score of 4.5 for the collagenase injection and 3.3 for the passive extension manipulation after anaesthetic treatment. There seems to be a relationship between a patient’s level of pain during injection and the likelihood that the patient will experience pain during manipulation [33]. A wrist block before a collagenase injection can reduce perceived pain and potentially increase patient satisfaction [34]. However, increased pain does not persist for a long time as the average VAS score was around 1 for the 7–365-day FU.

The satisfaction rate was high with a slight deterioration within 1 year, probably because of an increase in the flexion contracture and diminished subjective function (Table 2). This satisfaction rate is in line with the other authors [23, 18, 15, 30, 31] who also showed that patients are more satisfied with collagenase treatment than with placebo.

Many patients avoid surgical treatment because of their advanced age, coexisting medical conditions or fear of surgery. The availability of the collagenase treatment might mobilize a treatment-naive group of patients that would otherwise never present themselves or who are simply afraid to visit surgeon.

The recurrence rate ranges between 10 and 47% with the PIP joint being more prone to recurrence than the MCP joint [16, 17, 35]. However, only a minor percentage of patients who show a recurrence undergo further interventions [35, 17]. This is in concordance to the presented data, as only 2% of patients needed surgical treatment during the follow-up period. Surgical treatment was mainly conducted in the beginning of this observational study as experience was low with the use of Xiapex® leading to few secondary partial fasciectomy treatments. However, because of a potential lack of recorded data, further investigations are necessary to evaluate the long-term effectiveness of the collagenase treatment and the recurrence rate, which needs further treatment. In addition, the recurrence rate seems similar to surgical treatment (0–39% for partial fasciectomy and 50–58% for needle aponeurotomy) and the types of complications differ in comparison to collagenase injections [16]. Surgical complications included mainly nerve injuries, neuropraxia, arterial injury, and complex regional pain syndrome, all of which was either not or rarely seen in collagenase treatment. Tendon injuries, skin injuries and haematomas showed a lower incidence in surgical treatment and peripheral oedema, extremity pain, injection site pain, haemorrhage and swelling, tenderness, pruritus and lymphadenopathy were not reported after fasciectomy in comparison to collagenase treatment [16, 27].

Finally, no serious side effects, major complications or rare adverse events such as infections, anaphylactic shocks and tendon, ligament, nerve or vessel injuries were seen in this prospective observational study in a large cohort.

There are several limitations. The study design did not allow uniform follow-up dates for all patients. Physician could start, interrupt or end the treatment at their and their patient’s convenience, respectively. In many cases patients decided simply to stop further follow-up, presumably because of missing problems. Therefore, data for follow-up visits were missing especially in the long term.

Moreover, the follow-up period of a maximum of 1 year might be insufficient to evaluate the recurrence rate. We are planning to present longer follow-up data. Finally, a potential selection bias needs to be taken into account as physicians were not obliged to report their patients.

In conclusion, the injectable collagenase *Clostridium histolyticum* (Xiapex®) gives us an alternative option for surgery in the treatment of Dupuytren’s disease especially for patients suffering from diabetes or medical co-morbidities which increase perioperative complication risks. Xiapex® proved to be effective and safe with a comparable recurrence rate to partial fasciectomy. The outcome in the treatment of the MCP joint seems better than for the PIP joint, yet in recurrent cases a repeated injection is possible. Minor adverse events disappear within 30 days and the need for surgical treatment within 1 year seems very low (2%). No major complications or rare side effects were seen in this prospective observational study.
